# Rural-urban differentials in child body mass index over time

**DOI:** 10.1186/s12887-023-04241-5

**Published:** 2023-08-22

**Authors:** Senahara Korsa Wake, Temesgen Zewotir, Gizachew Gobebo Mekebo, Yemane Hailu Fissuh

**Affiliations:** 1 College of Natural and Computational Sciences, Ambo University, Ambo, Ethiopia; 2 School of Mathematics, Statistics and Computer Science, University of KwaZulu-Natal, Durban, South Africa; 3 College of Natural and Computational Sciences, Aksum University, Axum, Ethiopia

**Keywords:** Body mass index, Longitudinal data, Mixed-effect, Rate of changes

## Abstract

**Background:**

The body mass index is a simple index based on weight and height that can be used to screen children and adults for potential weight problems. The objective of this study was to investigate urban-rural variations in child BMI and its distribution from 2006 to 2016 in four low and middle-income countries.

**Methods:**

This study used data from the Young Lives prospective cohort study conducted in Ethiopia, India, Peru, and Vietnam to assess the BMI change for children aged 5 to 15 between 2006 and 2016. We adopted a mixed-effect model to analyze the data.

**Results:**

The study revealed substantial changes and rises in BMI in Vietnam, Peru, India, and Ethiopia between 2006 and 2016. Peru had the highest BMI changes in both urban-rural areas. A low BMI was observed in Ethiopia and India. Urban-rural differences had a significant role in determining BMI variation. In urban Ethiopia, the mean BMI increased from 14.56 kg/m^2^ to 17.52 kg/m^2,^ and in rural areas, it increased from 14.57 kg/m^2^ to 16.67 kg/m^2^. Similarly, in urban Vietnam, the BMI increased from 16 kg/m^2^ to 20.3 kg/m^2,^ and in rural areas, it increased from 14.69 kg/m^2^ to 18.93 kg/m^2^.

**Conclusions:**

The findings showed an increase in BMI changes in Ethiopia, India, Peru, and Vietnam from 2006 to 2016. Urban-rural differences have a significant contribution to determining BMI variation.

## Background

Plotting variations in height and weight to monitor growth is an essential and long-standing part of health maintenance in children and adolescents. Physical height and weight are crucial health-related factors. Body mass index (BMI) is a useful screening measure for children and adults to identify potential weight problems as it represents a simple index based on weight and height [1, 2]. BMI is calculated by dividing the weight in kilograms by the square of the height in meters. Epidemiology studies have identified high BMI as a well-known risk factor for several chronic illnesses such as cardiovascular disease, diabetes, cancer, chronic kidney disease, and a variety of musculoskeletal disorders [3–7]. Concerns about the economic and health impact of rising BMI have led to the inclusion of obesity among the global non-communicable disease targets, intending to limit the growth in obesity prevalence at its 2010 level by 2025 [8]. To enhance accountability for global non-communicable disease commitments, information on whether countries are on a path to meet this objective is required.

Child growth is identified as an essential measure of a population’s or country’s nutritional status [9–12]. The BMI is an important population-level measure of malnutrition status [1]. Underweight, stunting, wasting, and being overweight are all signs of malnutrition and influence children’s growth. Overweight and obesity are among the serious public health problems being faced worldwide today [13–15]. Previous research found an increase in BMI in many countries [16–18]. Although increases in mean BMI have been seen in many countries, the change may follow distinct changes in different countries and populations [19].

The World Health Organisation estimates that 1.6 billion people worldwide are overweight or obese [1]. Mean body mass index (BMI) scores must be evaluated not only for the entire population but also for various demographic groups in order to establish effective public health policies and interventions [2]. There have been worrying rises in mean BMI over the past few decades in numerous nations. Despite the fact that rises in the mean BMI have been noted in several nations, these changes may manifest differently in each nation. For instance, the mean BMI in the UK grew between 1980 and 1993 from 24.3 to 25.9 for males and from 23.9 to 25.7 for women [2]. There is also an increment in BMI in different countries [20, 21].

Understanding country-specific BMI changes and the potential differences between them is therefore important. Previous studies reported that health-related variables including age, physical activity, smoking, self-reported health status, and socio-demographic characteristics are all associated with BMI [20, 22].

Cross-sectional data are largely the source used in the extensive research on health disparities between rural and urban areas. This study used longitudinal data to look at differences in child BMI between rural and urban regions. Understanding longitudinal country-specific potential disparities across population groups in mean BMI changes over time is so critical. More precise information on which population groups have the highest increases in mean BMI over time may guide clinicians and public health in their preventive work as well as strategies. A lot has been written on the disparities in health between rural and urban areas in low- and middle-income countries. Despite the fact that changes in children’s BMI status over time have been reported in certain countries, low- and middle-income countries have received little comparison. We used the Young Lives data to estimate changes from 2006 to 2016 in mean BMI and urban-rural differentials in BMI changes among all four countries. This study aimed to examine the urban-rural variations in child BMI across time.

## Materials and methods

### Data source and ethical aspects

The data were collected as part of the Young Lives cohort study, which is a 15-year longitudinal study that looks at the changing nature of childhood poverty in four low- and middle-income countries: Ethiopia, India, Peru, and Vietnam. These four countries have a wide range of socio-economic and political features. The Young Lives cohort study thus collects data from these countries at both child-level and household levels to understand the causal effect of childhood poverty [23].

A multistage sampling technique was used for sample selection, with the first stage involving a selection of 20 sentinel locations from each country. Following the selection of 20 sentinel sites, households with children on average one-year age groups were chosen at random. Finally, within the designated sites,100 children were chosen at random [24]. The details about the sample and sampling techniques used in the Young Lives cohort study were discussed in the previous studies [25–29].

The anthropometric measurements of children were collected in 2006, 2009, 2013, and 2016 years [30]. This study considered children who had four anthropometric measurements from age 5 to 15 years. A total of 28,500 observations were obtained from 7125 children. Formal ethical approval for this study was obtained from the Young Lives study which was reviewed and approved by the Ethics Committee of Oxford University.

### Variables of the study

The continuous response variable was BMI, calculated for each individual as weight (kg)/height (${m}^{2}$). Measuring BMI is an adequate screening technique for finding an unusual weight-to-height ratio. Weight and height were measured for each child four times. Individuals with missing values for either height or weight were not considered. The assessment period comprised 4 categories: 2006, 2009, 2013, and 2016. The mean BMI was undertaken separately with explanatory variables like sex, age, and residential area for each country.

In the Young Lives data, loss to follow-up is quite rare. The loss to follow-up was the cause of the sample attrition. Between rounds 1 and 3, 72 children died in Ethiopia, 36 in India, 20 in Peru, and 11 in Vietnam [31]. Only children sampled at all four rounds of data collection and who have non-missing data on the dependent and independent variables are included in this study, yielding sample sizes of 7125 across all four countries. Furthermore, children with at least one missing value on the dependent and independent variables were excluded from this study. For instance, 7.3%, 4%, 6.5%, and 3.5% were excluded from Ethiopia, India, Peru, and Vietnam, respectively. As quantitative information, the anthropometric measurements of height in cm and weight in kg were taken. In the Young Lives sample, the wealth index serves as the main indication of the socioeconomic standing of households. It is built based on the standard of housing, accessibility to services, and possession of consumer goods [23].

### Data analysis

Descriptive statistics were used to assess the distribution of the explanatory variables and mean BMI for the explanatory variables by sex, rural-urban location, and year of assessment. To examine the rate of change in the mean BMI over the study period, a mixed-effect model of the mean BMI against age was estimated. This model has a high potential for measuring within-between variations in variables and can account for the correlation present in the mean BMI data [32, 33]. The details mathematical expression of a mixed-effect model is available from the first author’s studies [26, 27].

A fractional polynomial was adopted to approximate the nonlinear changes in mean BMI using power transformations of the time metric with integer and non-integer exponents [26, 27]. The power terms are selected from (-2, -1, -0.5, 0, 0.5, 1, 2, 3) [34, 35]. A mixed-effect model was used to analyse differences in child BMI across urban and rural areas over time after a time function representing nonlinear changes in BMI was determined. The data were analyzed using SAS version 9.4 software. A mixed-effect model [36, 37] can be formulated as

$${\varvec{y}}_{i}={\varvec{X}}_{i}\beta +{\varvec{Z}}_{i}{\varvec{b}}_{i}+{\epsilon }_{i}$$

where, ${\varvec{y}}_{\varvec{i}}$ is the ${(n}_{i}\times 1)$ vector of continuous outcome for the i^th^ individual, ${\varvec{X}}_{i}$ is an $({n}_{i}\times p)$ covariate matrix related to the fixed effects $\beta$, where $\beta ? {R}^{p\times 1}$, ${\varvec{Z}}_{i}$ is the $({n}_{i}\times q)$ design matrix related to random effects ${\varvec{b}}_{i}$, where ${\varvec{b}}_{i} ? {R}^{q\times 1}$, and ${\epsilon }_{i}$ is the ${(n}_{i}\times 1)$ within-individual error vector.

## Results

### Sample description and BMI distribution

**Fig. 1:**
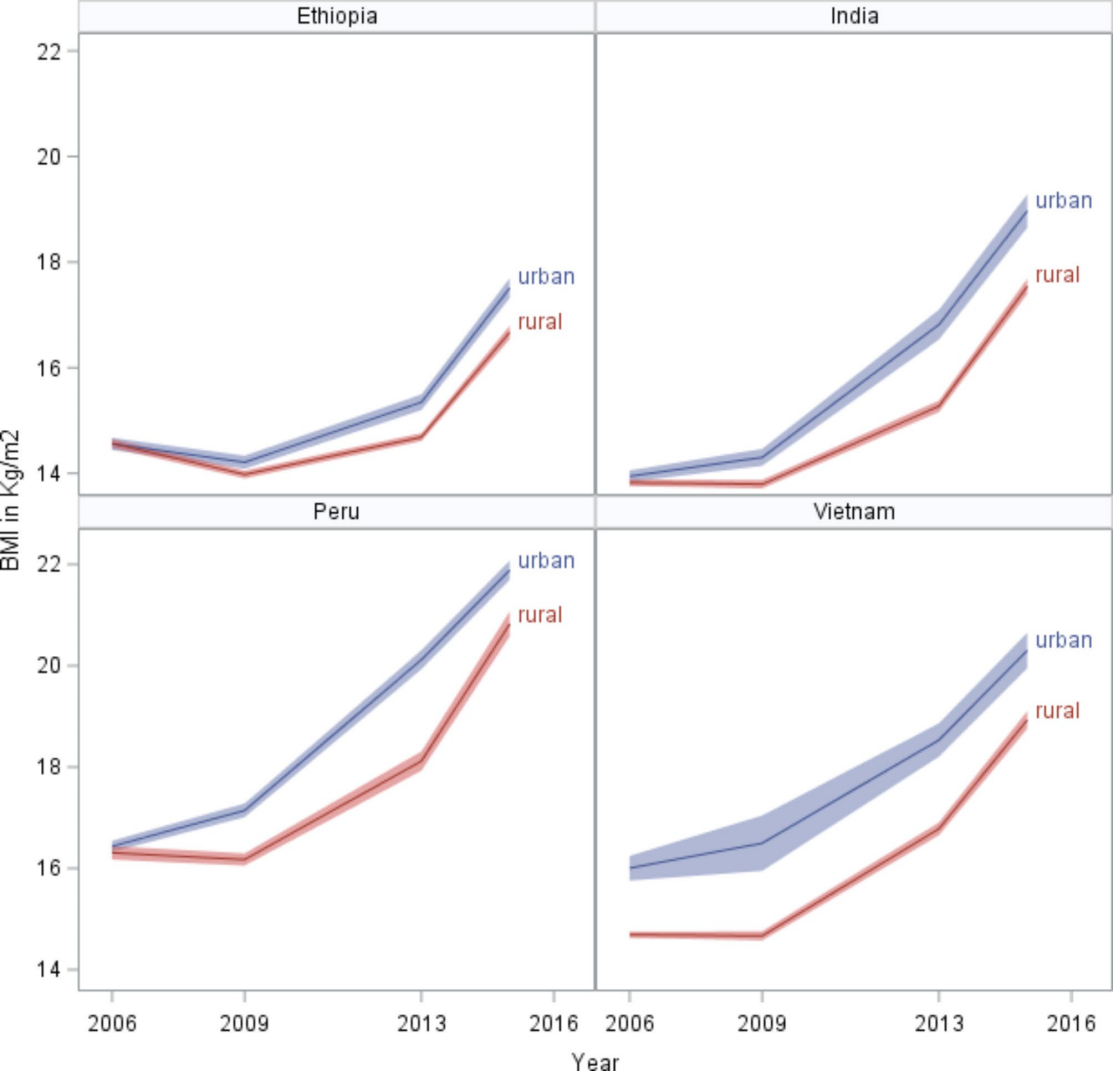
Changes in mean BMI over time by living area from 2006 to 2016

**Fig. 2:**
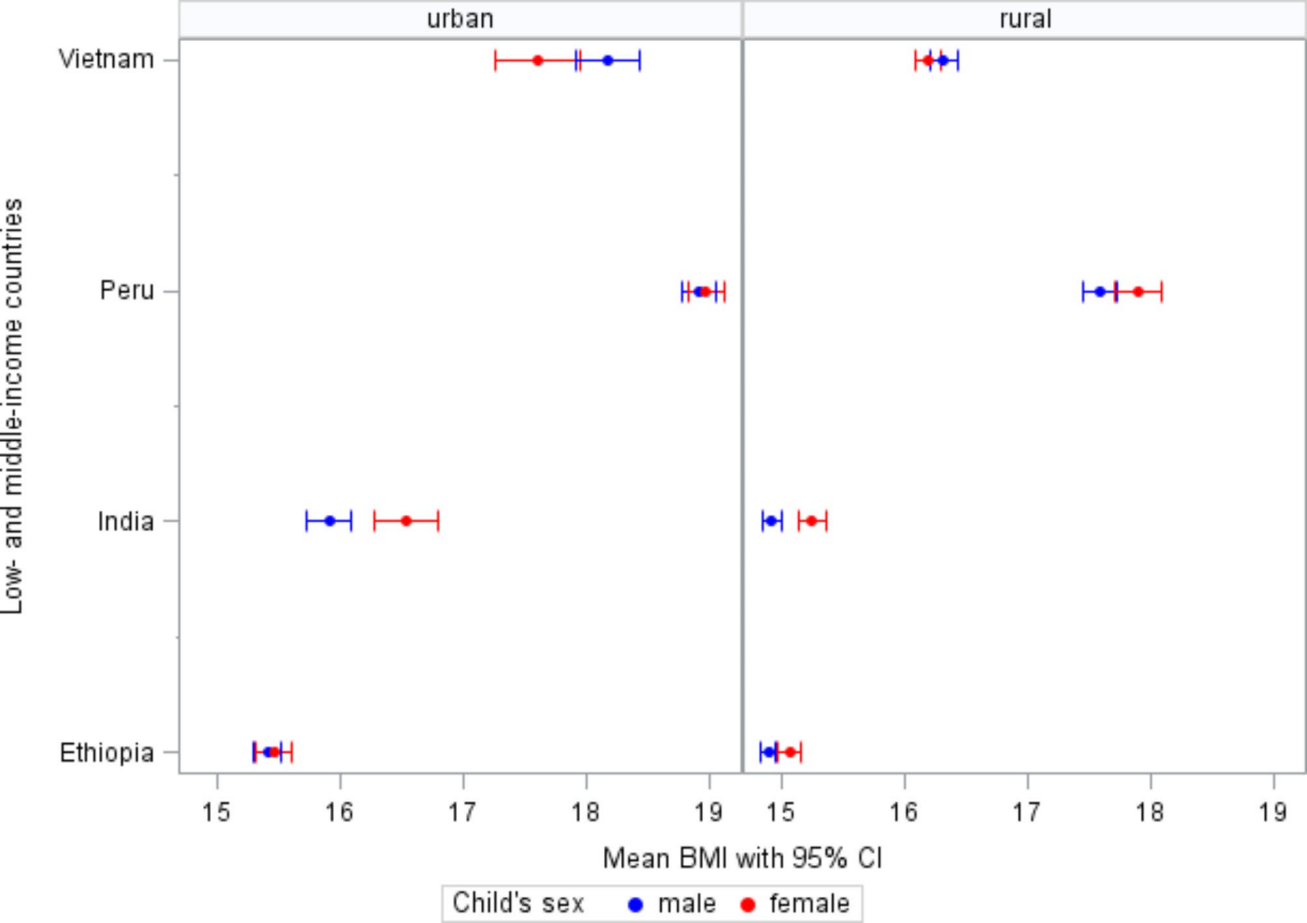
Changes in mean BMI over time by living area and sex from 2006 to 2016

The analysis involved a total of 7125 children: 37% were from urban areas and 63% were from rural areas. The descriptive statistics of the participants by year and living area were displayed in Table 1. In Table 1, the distribution of BMI from 2006 to 2016 is presented separately by urban-rural location. The mean BMI increased at different rates in both urban and rural regions across all four countries from 2006 to 2016 (Figs. 1 and 2). For instance, the mean BMI increased from 15.52 kg/m^2^ (95% CI: 15.44–15.60) to 20.16 kg/m^2^ (95% CI: 20.02–20.30) for urban children and from 14.58 kg/m^2^ (95% CI: 14.54–14.63) to 18.15 kg/m^2^ (95% CI: 18.05–18.24) for urban children in 2006 to 2016, respectively.

**Fig. 3:**
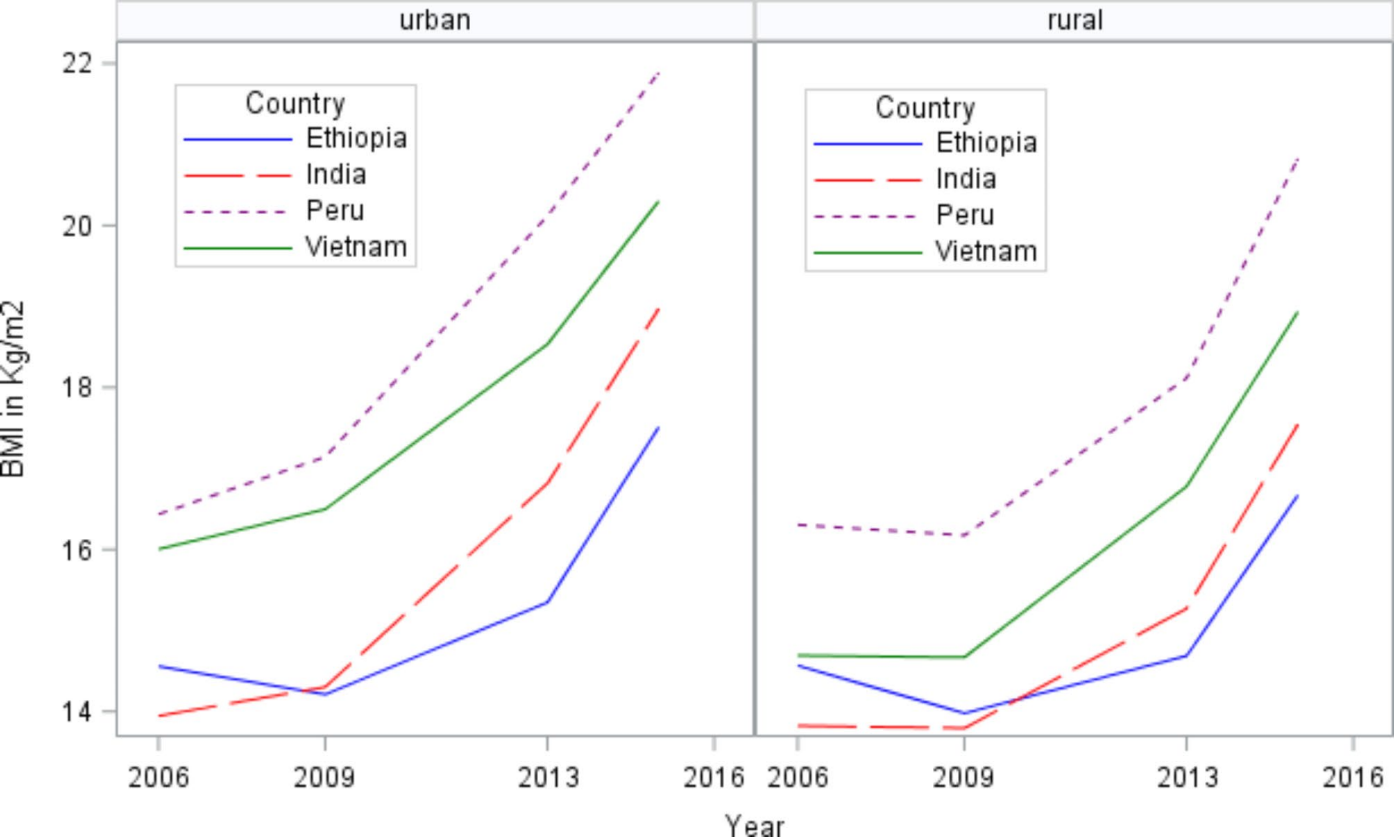
Changes in mean BMI over time by urban and rural areas in four countries from 2006 to 2016

The changes in mean BMI for urban-rural areas were not consistent across the four low and middle-income countries. Peru had the largest mean BMI in both urban-rural areas while Ethiopia had the lowest BMI (Fig. 3). The summary descriptive statistics of the study participants were presented in Table 1.

**Table 1:** Descriptive statistics of the study population by living area, count (percent) or mean (standard deviation)

Year (mean age )		Ethiopia		India		Peru		Vietnam	
		Urban	Rural	Urban	Rural	Urban	Rural	Urban	Rural
2006 (5)	BMI (mean)	14.56(1.41)	14.57(1.45)	13.95(1.23)	13.82(1.25)	16.44(1.94)	16.31(1.56)	16.00(2.33)	14.69(1.24)
	Height (mean)	105.16(5.30)	103.13(5.49)	105.48(5.14)	103.59(5.67)	106.17(5.88)	99.62(5.08)	108.27(5.52)	104.13(6.30)
	Weight (mean)	16.10(2.01)	15.50(1.97)	15.56(2.13)	14.84(1.83)	18.58(3.08)	16.18(1.82)	18.89(3.87)	15.96(2.11)
	Male (%)	308(52.60)	575(53.40)	256(55.30)	744(53.40)	626(51)	260(48.90)	190(53.20)	757(50.90)
	Female (%)	278(47.40)	502(46.60)	207(44.70)	649(46.60)	601(49)	272(51.10)	167(46.80)	730(49.10)
2009 (8)	BMI (mean)	14.21(1.54)	13.98(1.26)	14.15(1.80)	13.79(1.54))	17.14(2.45)	16.17(1.38)	16.50(5.31)	14.67(1.81)
	Height (mean)	122.60(6.40)	119.78(7.21)	121.35(6.34)	117.81(7.03)	121.37(5.75)	116.63(5.40)	124.59(6.03)	120.16(6.05)
	Weight (mean)	21.38(2.98)	20.08(2.76)	21.16(3.70)	19.14(2.55)	25.43(5.19)	22.05(2.90)	25.73(7.26)	21.26(3.67)
	Male (%)	310(52)	573(53.70)	255(55)	739(53.60)	642(50.70)	244(49.60)	193(53)	754(50.90)
	Female (%)	286(48)	494(46.30)	209(45)	641(46.40)	625(49.30)	248(50.40)	171(47)	726(49.10)
2013 (12)	BMI (mean)	15.35(1.85)	14.69(1.27)	16.83(3.31)	15.27(2.03)	20.12(3.43)	18.12(2.08)	18.60(3.37)	16.78(2.48)
	Height (mean)	142.83(7.43)	139.89(6.94)	142.53(8.29)	139.06(7.03)	144.17(7.41)	138.46(7.23)	148.69(8.04)	142.98(7.99)
	Weight (mean)	31.44(5.47)	28.84(4.09)	34.38(8.05)	29.73(5.77)	42.19(9.74)	34.99(6.61)	41.51(9.97)	34.59(7.47)
	Male (%)	318(52.20)	565(53.60)	293(56.30)	709(53.20)	657(50.70)	229(49.40)	191(52.60)	747(51.40)
	Female (%)	291(47.80)	489(46.40)	227(43.70)	624(46.80)	638(49.30)	235(50.60)	172(47.40)	707(48.60)
2016 (15)	BMI (mean)	17.52(2.04)	16.67(2.09)	19.04(3.83)	17.54(2.68)	21.89(3.47)	20.82(2.62)	20.30(3.52)	18.93(3.40)
	Height (mean)	158.00(7.52)	154.71(7.74)	156.9(8.24)	153.84(7.80)	157.73(7.45)	153.53(7.24)	160.74(7.98)	157.82(7.76)
	Weight (mean)	43.86(7.47)	40.09(6.95)	46.86(9.86)	41.66(7.90)	54.62(10.54)	49.26(8.16)	52.69(11.25)	47.20(8.82)
	Male (%)	322(52.10)	556(53.60)	305(55.80)	690(53.20)	666(50.60)	220(49.80)	210(53.60)	737(50.80)
	Female (%)	296(47.90)	482(46.40)	242(44.20)	608(46.80)	651(49.40)	222(50.20)	182(46.40)	715(49.20)

Note: BMI is a body mass index measured in $\frac{kg}{{m}^{2}}$

Figures 1 and 2 depict the urban-rural and sex differences in mean BMI in each country, respectively. Males had the highest mean BMI in all countries up to 2013 year, except in India up to the 2009 year, whereas females dominate the mean BMI at adult ages. However, the changes in mean BMI were largely similar across the four countries for males and females. In both sexes, Peru had the highest and Vietnam had the second highest mean BMI from 2006 to 2016.

Figure 3 shows the overall changes in mean BMI by urban-rural areas in four low- and middle-income countries. The urban-rural mean BMI was higher in middle-income countries (Peru and Vietnam) than in low-income countries (Ethiopia and India).

### Rates of changes in BMI

The changes of BMI over time was examined as a function of a child’s age. The visual inspection of the change shows a nonlinear change in BMI over time (Fig. 3). This led to the initial identification of the age function that most accurately captures this nonlinearity change in the mean BMI from 2006 to 2016. As a result, a second-order fractional polynomial with the logarithm of age (ln(age)) and linear of age are discovered to be the age function that best fits the change in BMI over time. Then, a fractional polynomial mixed-effect model was adopted to analyze the rates of change and rural-urban variations in BMI across time, and the results are summarized in Table 2.

**Table 2:** Estimates of rural-urban difference in BMI

Main Effect	Estimate	Standard Error	t-Value	Pr > |t|	95% CI	
					Lower	Upper
Intercept	24.4225	0.1894	128.93	< 0.0001	24.0512	24.7938
Height	-0.2206	0.0009	-256.02	< 0.0001	-0.2223	-0.2189
Weight	0.4534	0.0010	474.12	< 0.0001	0.4515	0.4553
Sex (Female)	0.0438	0.0099	4.42	< 0.0001	0.0244	0.0633
Residence area (Rural)	1.2935	0.1649	7.85	< 0.0001	0.9703	1.6167
**Country (Reference = Ethiopia)**						
India	-1.4123	0.2063	-6.85	< 0.0001	-1.8166	-1.0080
Peru	-0.9136	0.2163	-4.22	< 0.0001	-1.3376	-0.4896
Vietnam	-1.6683	0.2076	-8.04	< 0.0001	-2.0752	-1.2615
Age	0.3382	0.0179	18.95	< 0.0001	0.3033	0.3932
Log Age	4.8379	0.1580	30.62	< 0.0001	4.5283	5.1476
**Interaction Effect**						
Age*Rural	0.2691	0.0156	17.22	< 0.0001	0.2385	0.2998
Log Age*Rural	-1.8642	0.1401	-13.31	< 0.0001	-2.1388	-1.5897
Age*India	-0.0694	0.0197	-3.52	0.0004	-0.1080	-0.0308
Age*Peru	-0.3612	0.0207	-17.44	< 0.0001	-0.4018	-0.3206
Age*Vietnam	-0.3005	0.0198	-15.15	< 0.0001	-0.3394	-0.2616
Log Age*India	0.8937	0.1753	5.10	< 0.0001	0.5501	1.2372
Log Age*Peru	2.1861	0.1841	11.88	< 0.0001	1.8253	2.5468
Log Age*Vietnam	2.1526	0.1764	12.20	< 0.0001	1.8068	2.4984

The main and interaction effects of the covariates were investigated and the results are displayed in Table 2. The coefficients of sex and residence area were estimated to be 0.04 (p = < 0.0001) and 1.29 (p = < 0.0001), respectively. These suggest that both sex and urban-rural areas are statistically significant in determining a child’s BMI. The estimated country effects of India: -1.41, p = < 0.0001, Peru: -0.91, p = < 0.0001, and Vietnam: -1.67, p = < 0.0001 represent the mean difference in BMI between children in Ethiopia and (India, Peru, and Vietnam, respectively) at the intercept level. These values show that in comparison to Ethiopian children, children in India, Peru, and Vietnam had a lower significant mean BMI at intercept.

The interaction effect associated with age and urban-rural was positive and significant (Age*Rural: 0.27, p = < 0.0001). This suggests that there was a significant linear difference in BMI between urban and rural with higher linear changes in rural children. However, the interaction effect associated with ln(age) and urban-rural was negative and significant (ln(Age)*Rural: -1.86, p = < 0.0001) which implied rural children had a lower rate of change in BMI from 2006 to 2016 than urban children.

The interaction effect related to age and country ($\text{a}\text{g}\text{e}\times$ country) was negative and significant. In this regard, the significant interaction value (p = < 0.0001) revealed that the linear changes in BMI varied significantly among four low- and middle-income countries. Whereas, a positive interaction value of (($\text{l}\text{n}\left(\text{a}\text{g}\text{e}\right)\times$ country) shows that the rate of changes in BMI for children in India, Peru, and Vietnam was higher than that of Ethiopian children. However, the negative and significant linear change for the interaction of age and country suggests that children in Ethiopia had a higher linear change in BMI than children in India, Peru, and Vietnam (age $\times$ India: -0.07, p = 0.0.0004, age $\times$ Peru: -0.36, p = < 0.0001, age $\times$ Vietnam: -0.30 p = < 0.0001).

The estimates from the mixed-effect model shown in Table 2 support the findings that height and weight have a real effect on BMI [38]. The weight of children is positively and significantly associated with their BMI (weight: 0.45, p = < 0.0001). However, the height of children is negatively and significantly associated with their BMI (Height: -0.22, p = < 0.0001).

**Table 3:** Variance-Covariance Parameter Estimates of Random effects

Variance-covariance	Estimate	Standard Error	Z Value	p-value
Variance for Intercept	1.8251	0.04625	39.46	< 0.0001
Covariance for Intercept & slope	-0.1599	0.004178	-38.26	< 0.0001
Variance for Slope	0.0145	0.000405	35.76	< 0.0001
Residual	0.4213	0.005001	84.23	< 0.0001

The variance-covariance estimates of random effects given in Table 3 show BMI variations at slope. The statistical significance of the variance slope indicates that children’s mean BMI varied at the slope.

## Discussion

In this study, we analyzed the changes of BMI over time in four low and middle-income countries. To our knowledge, this was the first study to estimate changes in BMI over time by measuring the rate of change of the BMI and its distribution in Ethiopia, India, Peru, and Vietnam. Furthermore, the nutritional status and changes in height and weight of children in these countries had been studied previously [25, 26, 39–42]. Thus, the analysis of BMI changes reported in this study is not only more complete but also more methodologically sound and updated.

This study performed a longitudinal analysis to examine the BMI changes from 2006 to 2016 using longitudinal evidence obtained from the Young Lives cohort study in Ethiopia, India, Peru, and Vietnam. The nonlinear changes were observed in BMI over time. Thus, time transformation was performed to examine the nonlinear changes in BMI over time. As a consequence, a second-order fractional polynomial with logarithmic and linear time power terms was selected for fitting the nonlinear BMI over time. Finally, we adopted a linear mixed-effect model to analyze a nonlinear change in BMI over time.

The study found significant increases in BMI between 2006 and 2016 in all urban-rural areas of the countries. In urban Ethiopia, the BMI increased from 14.56 kg/m^2^ to 17.52 kg/m^2,^ and in rural areas, it increased from 14.57 kg/m^2^ to 16.67 kg/m^2^. In urban India, the BMI increased from 13.95 kg/m^2^ to 19.04 kg/m^2,^ and in rural areas, it increased from 13.82 kg/m^2^ to 17.54 kg/m^2^. In urban Peru, the BMI increased from 16.44 kg/m^2^ to 21.89 kg/m^2,^ and in rural areas, it increased from 16.31 kg/m^2^ to 20.82 kg/m^2^. Similarly, in urban Vietnam, the BMI increased from 16 kg/m^2^ to 20.3 kg/m^2,^ and in rural areas, it increased from 14.69 kg/m^2^ to 18.93 kg/m^2^. Although the rate of BMI increase varies between nations, our observations of a rising tendency over time are consistent with other studies [43].

The study identified that there is a significant variation in children’s BMI between urban and rural locations. Compared to children in urban areas, children in rural areas had higher BMI. This finding is consistent with the study done by Banach et al. (2022) [44] reported that BMI increased slightly more in rural than in urban areas in South Asia, sub-Saharan Africa, and some countries in central and eastern Europe.

In the four study countries, the rate of change in BMI varied. Peru had the greatest overall BMI changes in both urban and rural regions. In contrast, the rate of changes in BMI was low for children in Ethiopia. The study found that there were substantial differences in BMI changes between urban and rural areas. Our findings indicating an increasing changes in BMI over time are consistent with prior studies; however, the magnitude of the increase varies per country [2]. A longitudinal study conducted in the US [43] and Sweden [2] reported annual increases in BMI.

Previous studies on BMI change over time relied on cross-sectional [45, 46] or single-cohort studies [47, 48]. Cross-sectional studies make it difficult to distinguish whether observed weight gain represents actual age effects [2]. However, we used longitudinal data obtained from four measurements of the same individuals which allowed us to investigate variations in BMI by sex and urban-rural areas. An advantage of longitudinal study over cross-sectional study is the possibility to distinguish changes over time within and between groups [16, 32, 33, 49–51]. The Key strength of this study includes the follow-up of BMI changes in individuals for a long period together with the repeated measurements of BMI for each individual.

This study has also its own limitations. The study was limited to four low and middle-income countries, which may not be representative of all low- and middle-income countries. The association of BMI with pubertal stage is not included in this study. The previous study reported that BMI might be associated with pubertal stages [52]. As a result, more research is needed to address this constraints.

## Conclusion

The novelty of this study comes from the long follow-up individuals obtained from the sample of the Young Lives prospective cohort study. In conclusion, this study provides a comprehensive assessment of BMI changes in four low and middle-income countries. The findings showed a greater increase in BMI changes in all countries from 2006 to 2016. Urban-rural differences provide a significant contribution to determining BMI variations.

## Data Availability

The datasets analyzed during the current study are available in the Young Lives study repository, http://www.younglives.org.uk/.
